# Supplementary material for: Development of a computer-aided design software for the quantitative evaluation of aesthetic damage
Source: PLoS One. 2019 Dec 18;14(12):e0226322. doi: 10.1371/journal.pone.0226322 (PMC6919621; doi:10.1371/journal.pone.0226322)
Supplement: S1 Code site — 1. (ZIP) [file pone.0226322.s001.zip › [EESC_jr][Projeto]Relatorio_07_21/reports/templates_zip/gentelella-master.zip!/gentelella-master/production/tables.html]

Gentellela Alela! | 


Gentellela Alela!

Welcome,

## John Doe

  


### General

- Home 
  - Dashboard
  - Dashboard2
  - Dashboard3
- Forms 
  - General Form
  - Advanced Components
  - Form Validation
  - Form Wizard
  - Form Upload
  - Form Buttons
- UI Elements 
  - General Elements
  - Media Gallery
  - Typography
  - Icons
  - Glyphicons
  - Widgets
  - Invoice
  - Inbox
  - Calendar
- Tables 
  - Tables
  - Table Dynamic
- Data Presentation 
  - Chart JS
  - Chart JS2
  - Moris JS
  - ECharts
  - Other Charts
- Layouts 
  - Fixed Sidebar
  - Fixed Footer

### Live On

- Additional Pages 
  - E-commerce
  - Projects
  - Project Detail
  - Contacts
  - Profile
- Extras 
  - 403 Error
  - 404 Error
  - 500 Error
  - Plain Page
  - Login Page
  - Pricing Tables
- Multilevel Menu 
  - Level One- Level One
      - Level Two
      - Level Two
      - Level Two
    - Level One
- Landing Page Coming Soon

- John Doe
  - Profile
  - 50%
    Settings
  - Help
  - Log Out
- 6
  - John Smith
    3 mins ago

    Film festivals used to be do-or-die moments for movie makers. They were where...
  - John Smith
    3 mins ago

    Film festivals used to be do-or-die moments for movie makers. They were where...
  - John Smith
    3 mins ago

    Film festivals used to be do-or-die moments for movie makers. They were where...
  - John Smith
    3 mins ago

    Film festivals used to be do-or-die moments for movie makers. They were where...
  - **See All Alerts**


### Tables Some examples to get you started

Go!

## Basic Tables basic table subtitle

- - Settings 1
  - Settings 2

| # | First Name | Last Name | Username |
| --- | --- | --- | --- |
| 1 | Mark | Otto | @mdo |
| 2 | Jacob | Thornton | @fat |
| 3 | Larry | the Bird | @twitter |

## Stripped table Stripped table subtitle

- - Settings 1
  - Settings 2

| # | First Name | Last Name | Username |
| --- | --- | --- | --- |
| 1 | Mark | Otto | @mdo |
| 2 | Jacob | Thornton | @fat |
| 3 | Larry | the Bird | @twitter |

## Hover rows Try hovering over the rows

- - Settings 1
  - Settings 2

| # | First Name | Last Name | Username |
| --- | --- | --- | --- |
| 1 | Mark | Otto | @mdo |
| 2 | Jacob | Thornton | @fat |
| 3 | Larry | the Bird | @twitter |

## Boardered table Bordered table subtitle

- - Settings 1
  - Settings 2

| # | First Name | Last Name | Username |
| --- | --- | --- | --- |
| 1 | Mark | Otto | @mdo |
| 2 | Jacob | Thornton | @fat |
| 3 | Larry | the Bird | @twitter |

## Table design Custom design

- - Settings 1
  - Settings 2

Add class `bulk_action` to table for bulk actions options on row select

|  | Invoice | Invoice Date | Order | Bill to Name | Status | Amount | Action | Bulk Actions (   ) | | | | | | |
| --- | --- | --- | --- | --- | --- | --- | --- | --- | --- | --- | --- | --- | --- | --- |
|  | 121000040 | May 23, 2014 11:47:56 PM | 121000210 | John Blank L | Paid | $7.45 | View |  |  |  |  |  |  |  |
|  | 121000039 | May 23, 2014 11:30:12 PM | 121000208 | John Blank L | Paid | $741.20 | View |  |  |  |  |  |  |  |
|  | 121000038 | May 24, 2014 10:55:33 PM | 121000203 | Mike Smith | Paid | $432.26 | View |  |  |  |  |  |  |  |
|  | 121000037 | May 24, 2014 10:52:44 PM | 121000204 | Mike Smith | Paid | $333.21 | View |  |  |  |  |  |  |  |
|  | 121000040 | May 24, 2014 11:47:56 PM | 121000210 | John Blank L | Paid | $7.45 | View |  |  |  |  |  |  |  |
|  | 121000039 | May 26, 2014 11:30:12 PM | 121000208 | John Blank L | Paid | $741.20 | View |  |  |  |  |  |  |  |
|  | 121000038 | May 26, 2014 10:55:33 PM | 121000203 | Mike Smith | Paid | $432.26 | View |  |  |  |  |  |  |  |
|  | 121000037 | May 26, 2014 10:52:44 PM | 121000204 | Mike Smith | Paid | $333.21 | View |  |  |  |  |  |  |  |
|  | 121000040 | May 27, 2014 11:47:56 PM | 121000210 | John Blank L | Paid | $7.45 | View |  |  |  |  |  |  |  |
|  | 121000039 | May 28, 2014 11:30:12 PM | 121000208 | John Blank L | Paid | $741.20 | View |  |  |  |  |  |  |  |


Gentelella - Bootstrap Admin Template by Colorlib
